# The integration of Open3DTOOLS into the RDKit and KNIME

**DOI:** 10.1186/1758-2946-6-S1-P8

**Published:** 2014-03-11

**Authors:** Paolo Tosco, Nikolaus Stiefl, Greg Landrum

**Affiliations:** 1 Department of Drug Science and Technology, University of Turin, Torino, I-10125, Italy; 2 Novartis Institutes for Biomedical Research, Basel, CH-4002, Switzerland

## 

Open-source software is especially appealing to academics, since it permits implementing novel methods into existing code with little effort, while allowing full disclosure of the underlying science; the lack of license fees and the ease of dissemination via public repositories are additional plusses.

However, open-source has also been growing popular within large companies, which have recognized the value of code sharing to increase the pool of users (and therefore testers and reviewers), as well as to gather new ideas and contributions. In the pharmaceutical field, an outstanding example is represented by the RDKit [1], a BSD-licensed C++ cheminformatics toolkit with Python, Java, and C# bindings, originally developed at Rational Discovery and currently used and being actively developed within the Novartis Institutes for BioMedical Research, which contributes in-house code enhancements back to the open-source version.

To enable effective and widespread adoption and usage of open-source tools, it is helpful to make them available as plugins to well-recognized platforms. Herein we describe the integration of Open3DTOOLS (namely, Open3DQSAR [2] and Open3DALIGN [3]) into the RDKit and KNIME [4]. This task required the preliminary implementation and validation of the MMFF94(s) force field, upon which the Open3DTOOLS are based, in the C++ layer of the RDKit, followed by the extension of the RDKit API to enable molecular alignment, MIF computation and 3D-QSAR model building. Additionally, KNIME nodes were set up to allow access to Open3DTOOLS functionality within KNIME workflows.

We also present some test cases which illustrate the potential of these RDKit and KNIME extensions in the context of virtual screening and CADD.
